# My memories of the Benson-Henry institute for mind-body medicine

**DOI:** 10.1186/s13030-023-00270-9

**Published:** 2023-03-30

**Authors:** Mutsuhiro Nakao

**Affiliations:** Department of Psychosomatic Medicine, School of Medicine, International University of Health and Welfare, Narita, Japan

## Commentary letter

It is my great honor to introduce the review article [1] entitled “Mind-body medicine: A modern bio-psycho-social model 45 years after Engel” by Gregory Fricchione, MD, director of the Benson-Henry Institute for Mind Body Medicine and the Chester Pierce Division of Global Psychiatry, co-director of the McCance Center for Brain Health in the Department of Neurology at Mass General Hospital, and the Mind Body Medicine Professor of Psychiatry at Harvard Medical School. I also feel deeply saddened because Herbert Benson, MD, the director emeritus of the institute, passed away in 2022 before the publication of this article.

During the early years of my career at the institute, between 1998 and 2001, Dr. Benson was the founding director and consistently supported me by saying that “I am always on your side.” Dr. Fricchione was the research director in those days, and instructed me on how to write medical papers. We conducted a database study of outpatients participating in the medical symptom reduction program of the institute and reported the treatment effects of the program [2, 3]. At the farewell party, before my departure from Boston in the spring of 2001, Dr. Benson encouraged me to become an ambassador for the Mind-Body Medical Institute in Japan.

In 2021, I asked Drs. Benson and Fricchione to write a commentary letter in response to our editorial, [4] but Dr. Benson was already sick and bedridden. However, Dr. Frichhione remained in contact with me and completed this extraordinary review paper in 2023. The paper summarizes Engels’s previous works and proposes future research [1]. Based on Dr. Benson’s work, several models of stress managements have been integrated into mind-body medicine. Dr. Fricchione’s insights also provide useful guidance for the advancement of bio-psycho-social medicine.

## Data Availability

The data and materials were based on previously published information.

## Declarations

### Trial registration

The study was not registered as a trial.
